# Heterogeneity in Dyadic Coping Among Infertile Couples and Its Association with Depression and Fertility Quality of Life: A Latent Profile Analysis

**DOI:** 10.3390/healthcare14081031

**Published:** 2026-04-14

**Authors:** Xian Zhang, Yuetong Pei, Shanshan Dou, Chunhui Zhang, Yandan Duan, Jinling Gao

**Affiliations:** 1 School of Nursing and Health, Zhengzhou University, Zhengzhou 450001, China; zhangxian@zzu.edu.cn (X.Z.); peiyuetong0829@163.com (Y.P.); zhangchunhui0617@163.com (C.Z.); 2 Reproductive Medicine Center, The Third Affiliated Hospital of Zhengzhou University, Zhengzhou 450052, China; doushanshan1992@126.com

**Keywords:** infertile couples, dyadic coping, depression, fertility quality of life, latent profile analysis, assisted reproductive technology

## Abstract

**Highlights:**

**What are the main findings?**

- Four distinct dyadic coping profiles were identified among infertile couples undergoing assisted reproductive technologies.
- Several sociodemographic and clinical characteristics were significant predictors of profile membership.
- Both low and asymmetric dyadic coping profiles were associated with increased depressive symptoms and poorer fertility quality of life, with gender-specific vulnerability.

**What are the implications of the main findings?**

- These predictors may help identify couples who could benefit from further assessment of their coping patterns.
- Dyadic coping may serve as a protective resource for infertile couples to improve their psychological well-being and quality of life.
- The findings provide evidence to support couple-centered assessment and tailored psychosocial interventions in fertility care practices.

**Abstract:**

**Objective**: This study aimed to identify distinct dyadic coping profiles among infertile couples undergoing assisted reproductive technologies (ARTs) and to examine the associations between these coping profiles, depressive symptoms, and fertility quality of life (FertiQOL). **Methods**: A total of 271 infertile couples undergoing ARTs were recruited from a reproductive medicine center in Zhengzhou, China, and completed standardized self-report measures. Latent profile analysis was conducted to identify distinct dyadic coping profiles at the couple level. Multinomial logistic regression was used to examine sociodemographic and infertility-related predictors of profile membership. Differences in depressive symptoms and FertiQoL across profiles were analyzed using the Bolck–Croon–Hagenaars method. **Results**: Four dyadic coping profiles were identified: high-coping wife and low-coping husband (15.4%), low dyadic coping (20.1%), medium dyadic coping (31.5%), and high dyadic coping (33.0%). Couples in the high dyadic coping profile reported the lowest levels of depression and the highest level of FertiQoL. Women in the low dyadic coping profile reported the highest depressive symptoms, while men in the high-coping wife and low-coping husband profile demonstrated the highest depression among male partners. Sociodemographic factors (household registration, family income) and infertility characteristics (type of infertility, infertility duration) were significant predictors of profile membership. **Conclusions**: Dyadic coping among infertile couples undergoing ARTs is heterogeneous and differentially associated with depression and FertiQoL. Low and asymmetric dyadic coping represent high-risk profiles linked to poorer outcomes in both partners. These findings suggest that dyadic coping may serve as a protective resource for infertile couples to improve their psychological well-being and quality of life, highlighting the importance of incorporating dyadic coping assessment into routine care and providing couple-centered psychosocial interventions in fertility care practice.

## 1. Introduction

Infertility, clinically defined as failure to achieve pregnancy after 12 months of regular unprotected intercourse, affects approximately one in six of the global reproductive-age population [1]. Its rising prevalence, driven by delayed childbearing, environmental factors and lifestyle shifts, imposes multidimensional burdens including stigma, financial hardship, and compromised mental health [2,3]. Assisted reproductive technology (ART) has emerged as the cornerstone of infertility management, with approximately 2.5 million treatment cycles performed annually worldwide [4]. Despite technological advances, ART success rates remain modest. Moreover, the ART journey imposes considerable physical, emotional, and financial burdens on infertile couples [5,6], significantly impacting their psychosocial well-being.

As a shared stressor, the diagnosis of infertility and treatment process of ART often exacerbates great psychological distress among infertile couples [7]. Critically, accumulating evidence confirms a pattern of bidirectional negative emotional transmission between infertile patients and their partners [8] leading to relational strain [9] and decreased fertility quality of life (FertiQoL) for both partners within the couple dyad [10]. Furthermore, psychological distress, particularly depression, poses a significant threat to ART outcomes. Research demonstrates that such distress can adversely affect therapeutic efficacy by increasing treatment difficulties [11] and elevating the risk of treatment discontinuation [6,12], ultimately compromising reproductive success [13]. This compelling evidence underscores the critical necessity of optimizing psychosocial support into standard ART care protocols.

The Systemic Transactional Model (STM) (Bodenmann, 1995) provides a unified framework for understanding how couples cope with shared stressors such as infertility [14]. According to STM, external stress (e.g., infertility diagnosis and ART treatments) does not affect individuals in isolation. Instead, it triggers a dyadic stress-coping process, whereby one partner’s emotional distress and coping efforts influence—and are influenced by—the other partner’s responses, triggering shared appraisal and joint coping efforts [14]. Effective dyadic coping, characterized by supportive, collaborative, and well-coordinated partner responses [15], has been shown to buffer the negative impact of stress on both individual well-being and relationship quality [16,17]. For example, a recent study by Song et al. (2024) [9] confirms that dyadic coping mediates the relationship between fertility stress and marital quality among couples undergoing in vitro fertilization and embryo transfer (IVF-ET). And emerging evidence underscores that the coping strategies of one partner are associated with the psychological adjustment or maladjustment of the other partner at all periods of fertility treatment [18]. Understanding how couples dyadically cope with these shared stressors is therefore paramount for developing effective, couple-centered psychosocial support strategies in fertility care practice.

However, previous research has predominantly adopted a variable-centered approach (e.g., correlation, regression), which examines average relationships between coping strategies and outcomes across all individuals, implicitly assuming population homogeneity. While informative, this perspective overlooks the heterogeneity in couples’ coping patterns and the interdependence of couples’ psychosocial adaptation [18,19]. In contrast, a person-centered approach—specifically latent profile analysis (LPA)—assumes that populations are heterogeneous and can be classified into distinct subgroups (latent profiles) characterized by unique patterns of responses across multiple variables [20]. And LPA allows couples to be treated as a unified analytical unit and empirically identifies heterogeneous subgroups based on their shared response patterns [21]. This is also consistent with STM, which posits that individuals and their partners function as an interdependent system rather than as two separate entities when responding to shared stressors [14]. Extending the person-centered perspective to a dyadic focus allows us to capture not only congruent coping profiles (e.g., both partners being high or low in dyadic coping) but also incongruent or asymmetric profiles (e.g., high wife coping coupled with low husband coping), which have been largely overlooked in infertility research. To date, scholars have identified the associations between diverse dyadic coping profiles and psychosocial outcomes among couples affected by chronic conditions (e.g., cancer, stroke) [22,23]. Unlike chronic diseases, infertility and ART treatment represent significant dyadic stressors for couples of reproductive age, characterized by a coexistence of hope and psychological pressure, while also requiring greater shared decision-making and collaborative treatment engagement [24]. Therefore, the relationship between dyadic coping profiles in this unique context and both partners’ depressive symptoms as well as FertiQoL warrants in-depth investigation.

This study addresses these gaps by employing LPA to (1) identify latent profiles of dyadic coping among Chinese couples undergoing ART, (2) determine the distinct sociodemographic and clinical characteristics of each profile, and (3) explore associations between these profiles and both partners’ depression and FertiQoL. This analytical approach can reveal different potential categories of dyadic coping, providing new perspectives for theoretical development and targeted psychosocial interventions for infertile couples undergoing ART.

## 2. Materials and Methods

### 2.1. Study Setting and Participants

Between March 2024 and June 2024, a convenience sample of infertile couples who were receiving ART treatment was recruited from the reproductive medicine center of a tertiary hospital in Zhengzhou, China. The inclusion criteria were as follows: ① legally married; ② at least one spouse clinically diagnosed with infertility in accordance with the WHO definition; ③ scheduled to receive ART treatments, including artificial insemination (AI), in vitro fertilization and embryo transfer (IVF-ET), intracytoplasmic sperm injection (ICSI), and preimplantation genetic testing (PGT); ④ able to read and write fluently in Chinese; ⑤ able to provide informed consent and participate voluntarily. The exclusion criteria for both partners were as follows: ① withdrawing from the study after submission; ② having other serious physical illnesses; ③ being diagnosed with a mental disorder or having a history of mental disorders.

The sample size was determined based on multiple considerations. First, drawing on previous studies of dyadic coping profiles in couples [22,23], we anticipated a 3- to 5-class solution. To ensure adequate representation of each subgroup, a minimum of 50 couples per class was required [25], yielding a preliminary target of 250 couples. Second, according to Kendall’s rule of thumb for sample size estimation, the total sample size should be at least 5 to 10 times the number of independent variables [26]. Given that 15 independent variables were included in the planned analyses, a minimum of 75 to 150 couples was indicated. Finally, accounting for an anticipated 15% attrition or invalid response rate, the target sample size was set at 294 couples. In our study, 300 couples were accessed, with 14 couples refused due to lack of interest in the investigation. And after excluding questionnaires filled out only by wives or husbands (n = 12) or with consistent answers on their completed questionnaires (n = 3), 271 pairs of valid questionnaires were collected.

### 2.2. Measures

#### 2.2.1. Sociodemographic and Clinical Characteristics

A self-designed data sheet was used to collect couples’ sociodemographic and clinical characteristics. Sociodemographic data include age, marital duration, household registration, education level, employment status, and monthly household income per capita. Clinical data on primary etiology of infertility, type of infertility, infertility duration (years), ART treatment history (cycle numbers), and current treatment regimen (AI/IVF-ET/ICSI/PGT) were retrieved from medical records.

#### 2.2.2. The Chinese Version of the Dyadic Coping Inventory (C-DCI)

The Dyadic Coping Inventory was developed by Gmelch et al. (2008) [27] to measure couples’ joint responses and coping styles in response to stressful events. In this study, the Chinese version (C-DCI) adapted by Xu et al. (2016) [28] was used. The C-DCI consists of 31 items across five dimensions: stress communication (4 items), supportive coping (10 items), delegated coping (4 items), negative coping (8 items), and common coping (5 items). Each item is scored using a 5-point Likert scale (1 = “Very rarely” to 5 = “Very often”), with items in the dimension of negative coping reversely scored. A higher score reflects better dyadic coping. The C-DCI demonstrated good model fit and reliability (α = 0.74~0.93) [28]. Cronbach’s α coefficients were 0.953 (wives) and 0.952 (husbands) for spouses in this study, respectively.

#### 2.2.3. Patient Health Questionnaire-9 (PHQ-9)

Depressive symptoms were measured using the PHQ-9 [29], a 9-item instrument aligned with DSM-IV diagnostic criteria. Items are scored 0 (“Not at all”) to 3 (“Nearly every day”), with total scores categorized as: 0–4: no depression, 5–9: mild, 10–14: moderate, 15–19: moderately severe, 20–27: severe. The Chinese version adapted by Wang et al. (2014) [30] showed good reliability and validity. Current Cronbach’s α coefficients were 0.871 (wives) and 0.899 (husbands).

#### 2.2.4. The Fertility Quality of Life Tool (FertiQoL)

The FertiQoL tool is an international instrument to measure quality of life in men and women experiencing fertility problems [31]. The Chinese version of FertiQoL, which can be downloaded from http://www.fertiqol.org/ (accessed on 28 September 2023), was used in this study. This instrument is divided into two primary modules: a Core FertiQoL module (24 items) that measures the emotional, mind/body, relational, and social impact of infertility, and a Treatment FertiQoL module (10 items) that assesses perceptions of the treatment environment and its tolerability. This tool also includes two general health items. Responses are provided on a 5-point Likert scale, ranging from 0 (Very dissatisfied) to 4 (Very satisfied). Raw scores were converted to a 0–100 standardized score, with higher values representing a better quality of life. The Chinese version has previously shown robust psychometric properties [32]. And in the current sample, its reliability was excellent for both wives (Cronbach’s α = 0.921) and husbands (Cronbach’s α = 0.910).

### 2.3. Ethical Considerations and Procedures

This study was approved by the Institutional Review Board of Zhengzhou University (Approval No. ZZUIRB2023-304) on 8 December 2023. Approval to conduct the investigations was also obtained from the reproductive medicine center of the selected hospital. Two trained nursing students were responsible for collecting data. During couples’ waiting time for further assessment in the fertility clinic, both partners who met the inclusion criteria were invited to participate in the study. All participants provided informed consent prior to survey commencement, with explicit information about study aims, voluntary participation, confidentiality, and withdrawal rights. Upon obtaining informed consent, each couple was asked to fill in the questionnaires separately from each other. And the questionnaires were pre-coded so that couples in a dyad could be aligned for data entry. To express gratitude for their participation, we provided each couple who completed the survey with a small gift (a notebook valued at approximately 10 RMB). Data confidentiality was strictly maintained throughout the research process. All collected data were anonymized and stored on a password-protected server accessible only to the research team. Data analysis was performed using de-identified datasets to ensure participant privacy.

### 2.4. Statistical Analysis

Data analysis was conducted using SPSS version 22.0 and Mplus version 8.3. Given the use of self-report measures in this study, Harman’s single-factor test was employed to examine the common method bias. The results revealed that the first unrotated factor accounted for 26.52% of the total variance for wives and 25.25% for husbands, both below the recommended threshold of 40%, indicating the absence of obvious common method bias [33]. Descriptive statistics were used to summarize the sociodemographic and infertility-related information. Differences in depression scores between partners within infertile couples were assessed using Wilcoxon signed-rank tests. Paired-sample t-tests evaluated gender differences in dyadic coping and FertiQoL scores.

Latent profile analysis (LPA) was performed to identify dyadic coping profiles among infertile couples. The dyadic coping indicators entered into the LPA were the dimension scores of both partners on the Chinese version of the Dyadic Coping Inventory (C-DCI), including stress communication, supportive dyadic coping, delegated dyadic coping, common dyadic coping, and negative dyadic coping. The LPA was conducted at the couple level, treating the wife’s and husband’s scores as parallel indicators within the same model, allowing the identification of profiles characterized by distinct combinations of both partners’ coping patterns. Seven statistics were used to determine the number of latent profiles: the Akaike information criterion (AIC), the Bayesian information criterion (BIC), adjusted BIC (aBIC), the Lo–Mendell–Rubin likelihood ratio test (LMRT), the bootstrap likelihood ratio test (BLRT), entropy, and the minimum class size. Model selection followed established guidelines combining statistical fit indices with interpretability. Lower values of AIC, BIC, and aBIC indicate better fit [34]. A significant LMRT and BLRT result (*p* < 0.05) indicates that the model with k profiles fits better than that with k − 1 profiles. Entropy values > 0.80 indicate high classification accuracy [34,35]. Additionally, each retained profile should comprise at least 5% of the total sample to ensure clinical relevance [36]. Preference was given to the model with lower AIC, BIC, and aBIC values, higher entropy, and significant LMRT and BLRT results. When fit indices suggested multiple plausible solutions, the final model was selected based on simplicity, theoretical interpretability, and clinical relevance, ensuring that each profile contained a sufficient sample size (e.g., >5% of the total sample).

Univariate analyses, including one-way ANOVA, the Kruskal–Wallis H test and the chi-squared test, were used to identify the differences in the sociodemographic and clinical variables across the profiles. Subsequently, variables with a significance level of *p* < 0.10 in the univariate analyses were entered into a multinomial logistic regression model to identify potential predictors of latent profile membership. The Bolck–Croon–Hagenaars (BCH) method was employed to compare couples’ depression and FertiQoL across latent profiles [36]. This approach accounts for classification uncertainty by weighting cases based on posterior membership probabilities, thereby reducing estimation bias, and is recommended for evaluating auxiliary variables in LPA [37].

## 3. Results

### 3.1. Participant Characteristics

The study enrolled 271 infertile couples with a mean age of 32.08 ± 4.71 years (range: 21–47) for wives and 32.96 ± 4.54 years (range: 22–48) for husbands. The marital duration ranged from 1 to 23 years. Over 70% of participants resided in rural areas. Higher education attainment (college degree or above) was observed in 61.6% of wives and 54.5% of husbands. Primary infertility was reported by 128 couples (47.2%). The majority of couples (82.7%) underwent IVF-ET or the derived advanced technologies as their primary treatment regimen. Approximately two-thirds of participants (65.7%) were undergoing their first ART cycle. See Table 1 for detailed information.

### 3.2. Depression, Dyadic Coping and Fertility Quality of Life Scores Between Infertile Couples

Among infertile couples, the mean PHQ-9 scores were 4.45 (SD = 4.18) in wives and 4.51 (SD = 4.20) in husbands, with depression rates (PHQ-9 ≥ 5) of 43.2% (n = 117/271) and 42.4% (n = 115/271), respectively. Wives exhibited a mean dyadic coping score of 112.38 (SD = 15.94), while husbands scored 111.62 (SD = 16.63). The FertiQoL score was 66.83 (SD = 13.44) for wives and 68.43 (SD = 13.38) for husbands. There were no statistically significant differences between these variables (all Ps > 0.05). Detailed results are presented in Table 2.

### 3.3. Latent Profiles for Dyadic Coping

As shown in Table 3, the AIC, BIC, and aBIC values progressively decreased with increasing class numbers. And the entropy values exceeded 0.80 across all 2- to 5-class models. Although the 5-class model achieved the highest entropy (0.882), it was rejected due to a non-significant LMRT *p*-value (>0.05) and a smallest class proportion of 3% (n = 9), which fell below the minimum subgroup threshold of 5% recommended by Nagin (2005) [36]. Considering the fitting indices and theoretical interpretability, the 4-class model was selected as optimal. This indicates four distinct dyadic coping profiles among infertile couples undergoing ART. The average probability of each subgroup of infertile couples belonging to the profile ranged from 91.9% to 94.3% (see Supplementary Table S1), indicating that the results of the four-profile model are reasonable.

**Figure 1:**
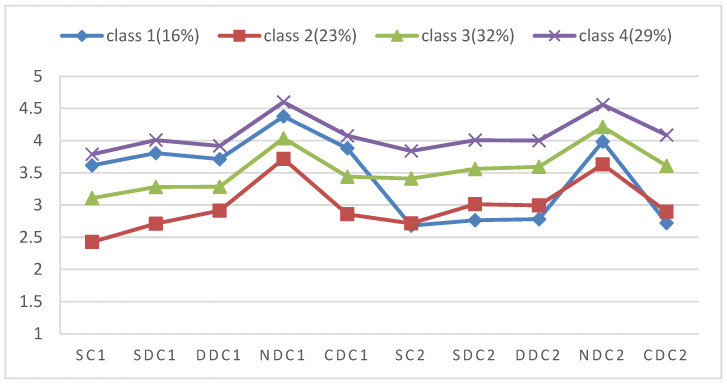
Latent profile model of dyadic coping based on dimension scores among infertile couples undergoing ART. Note: Class 1: high-coping wife and low-coping husband group, Class 2: low dyadic coping group, Class 3: medium dyadic coping group, Class 4: high dyadic coping group. SC1, SDC1, DDC1, NDC1, and CDC1 represent stress communication, supportive dyadic coping, delegated dyadic coping, negative dyadic coping, and common dyadic coping for wives, respectively. SC2, SDC2, DDC2, NDC2, and CDC2 represent the same constructs for husbands.

The four-class model from the LPA is depicted graphically with the mean scores of DCI dimensions in Figure 1. Class 1, consisting of 16% of the infertile couples (n = 42), was labeled the high-coping wife and low-coping husband group, with wives reporting relatively high levels of dyadic coping (M = 4.53, SD = 0.69) and husbands showing low levels across almost all dimensions (M = 2.70, SD = 0.61). Class 2 (n = 62, 23%) was labeled the low dyadic coping group in which both wives (M = 2.39, SD = 0.52) and husbands (M = 2.47, SD = 0.51) showed low levels of dyadic coping. Class 3 (n = 88, 32%), characterized by average levels on almost all DCI dimensions, was labeled the medium dyadic coping group. Finally, Class 4 (n = 79, 29%) had the highest scores of all DCI dimensions and was labeled the high dyadic coping group.

### 3.4. Multinomial Logistic Regression Analyses of Factors Affecting the Potential Categories of Dyadic Coping in Infertile Couples Undergoing ART

A multinomial logistic regression model was performed to explore the potential predictors of profile membership, with different dyadic coping profiles as the dependent variable, and the sociodemographic and clinical characteristics that were statistically significant in the univariate analysis (with a selection criteria of *p* < 0.1) as independent variables (see Supplementary Table S2). Multicollinearity among independent variables was identified using SPSS. The variance inflation factor (VIF) of all measured variables was below 5 (ranging from 1.093 to 1.613), and tolerance values were above 0.10 (ranging from 0.620 to 0.915), confirming the absence of multicollinearity. Specific results are shown in Table 4.

Class 4 (high dyadic coping group) served as the baseline reference category, with odds ratios (ORs) used as effect size estimates. Compared to couples in Class 4, a wife with a rural household registration, a husband with an urban household registration, and couples experiencing secondary infertility were susceptible to being included in Class 1 (high-coping wife and low-coping husband group). Couples with lower monthly household income per capita and a longer duration of infertility were more likely to belong to Class 2 (low dyadic coping group).

When using Class 2 (low dyadic coping group) as the baseline reference category, husband having urban household registration and couples with secondary infertility were found to have an elevated likelihood of belonging to Class 1 (high-coping wife and low-coping husband group). Couples with shorter duration of infertility were more likely to belong to Class 3 (medium dyadic coping group).

### 3.5. Comparisons of Depression and Fertility Quality of Life Among Subgroups

Differences in the PHQ and FertiQoL scores for the four dyadic coping subgroups were analyzed by the BCH method (see Table 5).

With regard to women’s outcomes, the overall tests showed there were significant mean differences in women’s depressive symptoms (χ^2^ = 33.88, *p* < 0.001) and FertiQoL (χ^2^ = 50.05, *p* < 0.001) across the four profile groups. The group comparisons further indicated that women in the high dyadic coping subgroup (Class 4) reported higher levels of FertiQoL and lower levels of depressive symptoms compared to women in other subgroups. Additionally, women in the low dyadic coping subgroup (Class 2) demonstrated the highest levels of depressive symptoms among the four subgroups. Both women in the low and medium dyadic coping subgroup (Class 2 and Class 3) had relatively lower levels of FertiQoL.

With regard to men’s outcomes, the overall tests also showed significant mean differences in depressive symptoms (χ^2^ = 20.97, *p* < 0.001) and FertiQoL (χ^2^ = 58.95, *p* < 0.001) across the four profile groups. Men in the high dyadic coping subgroup (Class 4) reported higher levels of FertiQoL and lower levels of depressive symptoms compared to men in other subgroups. Different from the comparison results for women, men in the high-coping wife and low-coping husband subgroup (Class 1) demonstrated the highest levels of depression symptoms among the four profile groups. In addition, both men in the high-coping wife and low-coping husband subgroup (Class 1) and the low dyadic coping subgroup (Class 2) showed lower levels of FertiQoL compared to men in the medium and high dyadic coping subgroups.

## 4. Discussion

This study innovatively applied latent profile analysis to examine heterogeneity in dyadic coping among infertile couples undergoing ART and to clarify how distinct dyadic coping profiles are associated with depressive symptoms and FertiQoL. By conceptualizing infertility as a shared dyadic stressor and treating the couple as the unit of analysis, our findings extend existing variable-centered research and provide nuanced evidence for stratified, couple-centered psychosocial interventions.

### 4.1. Dyadic Coping Heterogeneity in Infertile Couples

Using latent profile analysis, four distinct dyadic coping profiles were identified: high-coping wife and low-coping husband group, low dyadic coping group, medium dyadic coping group, and high dyadic coping group. This result underscores substantial heterogeneity in how couples jointly manage infertility-related stress, which would likely remain obscured using traditional mean-based approaches. As expected, 84% of couples adopted similar dyadic coping styles, as indicated by closely aligned scores across all dyadic coping dimensions, supporting the view that most couples treat infertility as a shared challenge [9]. These findings were partially congruent with previous studies on dyadic coping in chronic illness contexts [22,23] and underscore the necessity of involving both partners in targeted interventions.

Notably, the identification of a “high-coping wife and low-coping husband” profile reflects a pattern of dyadic incongruence that has been insufficiently explored in infertility research. Such asymmetry may reflect gendered coping roles within the sociocultural context of infertility, where women often assume primary responsibility for treatment engagement and emotional regulation [38], while male partners may adopt more avoidant or withdrawn coping strategies [19]. Crucially, dyadic coping is not merely the sum of two individuals’ coping efforts, but rather a relational process shaped by interactional dynamics [14]. Emerging evidence showed the congruence between partners in their stress appraisal and coping efforts directly shapes psychological and relational well-being [39,40]. Thus, clinical interventions should prioritize fostering open stress communication and collaborative treatment engagement within a supportive environment, thereby enhancing dyadic coping and promoting optimal outcomes for infertile couples.

### 4.2. Dyadic Coping Profiles, Depression, and Fertility Quality of Life

Couples in the high dyadic coping profile reported the lowest levels of depressive symptoms and the highest levels of FertiQoL across both partners. Conversely, couples in the low dyadic coping profile reported significantly higher levels of depression and lower levels of FertiQoL, consistent with previous research [18,41]. These findings provide robust evidence for the protective role of effective dyadic coping in the context of ART, consistent with a recent systematic review by Santamaría-Gutiez et al. (2025) [16], which concluded that positive dyadic coping may significantly enhance mental health, couple adjustment and satisfaction, as well as quality of life in couples undergoing ART.

Importantly, the asymmetric coping profile, characterized by a high-coping wife and low-coping husband, was associated with adverse outcomes comparable to, or in some cases worse than, those in low dyadic coping profile. This aligns with the stress and coping in couples model proposed by Pasch & Sullivan (2017) [42], which posits that incompatible dyadic approaches to managing infertility (i.e., mismatched appraisals and coping efforts) increase the likelihood of negative communication patterns and relationship deterioration. When one partner’s coping is not reciprocated, the protective function of dyadic coping may be undermined [39]. In this study, the low-coping husband may perceive their wife’s active coping not as support but as pressure, leading to feelings of inadequacy or of being a “failed supporter”, which in turn fuels depressive symptoms [18]. These findings reinforce the importance of assessing within-dyad discrepancies rather than focusing solely on overall coping levels. And men’s dyadic coping strategies should be a specific target of infertile couples’ interventions. In this context, couple-based counseling may offer particular benefits by emphasizing communication skills, mutual support and empathy, as well as shared coping strategies [43].

### 4.3. Predictors of Dyadic Coping Profile Membership

Several sociodemographic and clinical characteristics were associated with profile membership, including household registration status, monthly household income per capita, type of infertility, and infertility duration. Lower monthly income per capita and longer infertility duration predicted membership in the low dyadic coping profile, suggesting that cumulative treatment-related economic burden and time pressure may erode dyadic coping resources over time [6,44]. Specifically, our results confirm prior observations by Moura-Ramos et al. (2016) [45] that a longer duration of infertility was associated with a reduction in the protective effects of all coping strategies, which in turn negatively affected the couple’s dyadic adjustment. Additionally, discrepant urban–rural registration between partners (i.e., urban husbands and rural wives) emerged as a predictor of asymmetric coping patterns, potentially reflecting differences in health literacy, access to social resources, and gendered role expectations [46].

Notably, our findings reveal a significant gender disparity in dyadic coping among couples experiencing secondary infertility, where wives exhibit proactive coping while husbands demonstrate low engagement. This pattern is largely shaped by the interplay of traditional gender roles and the unique stressors of secondary infertility. Fertility problems are mostly the concern of women [47]. Meanwhile, wives, often adhering to the “emotional gatekeeper” role, proactively seek solutions and express emotions, consistent with coping strategies observed in female partners under fertility stress [19]. Conversely, for male partners with secondary infertility, their self-concept as fathers is relatively intact, allowing them to redirect their commitment to the “provider role” related social concerns rather than emotional engagement in the treatment process [47], which is paramount for the urban husbands.

The observed associations between sociodemographic factors and dyadic coping profiles suggest potential links to sociocultural dynamics, consistent with findings by Zhao et al. (2025) [48]. However, the cross-sectional design and the absence of direct measures of gender role attitudes or cultural norms preclude definitive conclusions. Rather than providing direct evidence for underlying sociocultural mechanisms, these findings should be interpreted as hypothesis-generating. Future research incorporating validated measures is needed to clarify the pathways linking these factors to dyadic coping profiles. Nevertheless, the findings highlight that dyadic coping is embedded within broader social and structural contexts, underscoring the need for culturally and contextually sensitive assessments and tailored interventions.

### 4.4. Clinical Implications and Study Limitations

The findings have substantial clinical implications, advocating for a paradigm shift from individual-focused to couple-centered psychological care in fertility practice. First, routine psychosocial assessment should move beyond individual distress screening to include evaluation of dyadic coping patterns within couples, which may help clinicians identify couples at elevated risk for maladaptive coping profiles. Second, couple-based interventions should be tailored according to coping profiles, with particular attention to couples characterized by low or imbalanced dyadic coping. For couples with low dyadic coping, particularly in the context of prolonged infertility, structured communication and coping enhancement interventions that rebuild shared coping strategies and mutual understanding are warranted [49]. For couples exhibiting asymmetric coping, dyadic coping training focused on validating the less-engaged partner’s emotional experience and fostering a balanced support exchange may be beneficial. In addition, couple-based counselling integrated into ART settings can serve as a platform to address coping disparities early and enhance both partners’ psychological adjustment and treatment experience [43].

Several limitations must be acknowledged. First, the cross-sectional design precludes inferences of causality. Future longitudinal studies are needed to delineate how these profiles evolve over the course of treatment. Second, our data were based on self-report measures, which may be subject to social desirability and common method bias. Although Harman’s single-factor test suggested that common method bias was not a serious concern, future studies should incorporate objective measures or multi-source data where feasible. Third, the single-site convenience sampling may introduce selection bias, as couples seeking ART at a specialized hospital may differ systematically from the broader infertile population. Consequently, the dyadic coping profiles identified in this sample may not be directly generalizable to community-based infertile populations. Finally, the study was conducted within the Chinese cultural context. Given that cultural and gender differences across the globe can influence how couples’ coping behavior affects relationship outcomes [50], the generalizability of these findings warrants further investigation.

## 5. Conclusions

This study reveals infertile couples undergoing ART exhibit distinct dyadic coping profiles that are differentially associated with depressive symptoms and FertiQoL. High dyadic coping may serve as a protective relational resource, whereas low or imbalanced coping appears to be associated with heightened psychosocial vulnerability. Notably, our findings also suggest that dyadic coping is embedded within broader social and structural contexts, highlighting the role of external determinants in couples’ coping processes. By adopting a person-centered dyadic perspective, this research extends current understanding of dyadic coping mechanisms in infertility and provides an empirical foundation for developing tailored, couple-focused psychosocial interventions. Incorporating dyadic coping assessment into routine fertility care, along with targeted interventions such as couple-based counselling, dyadic coping training, and structured communication interventions, may contribute to improved psychological well-being and quality of life among infertile couples.

## Figures and Tables

**Table 1:** Participant characteristics (n = 271 pairs).

Item	Wife (n = 271)	Husband (n = 271)
Age (years)	32.08 ± 4.71 (21–47)	32.96 ± 4.54 (22–48)
Years of marriage	5.71 ± 3.99 (1–23)	
Household registration		
Urban	77 (28.4)	74 (27.3)
Rural	194 (71.6)	197 (72.7)
Education level		
High school and below	104 (38.4)	126 (46.5)
College and above	167 (61.6)	145 (54.5)
Employment status		
Full time	117 (43.2)	122 (45.0)
Part time	154 (56.8)	149 (55.0)
Monthly household income per capita		
<3000 RMB	61 (22.5)	
3000–4999 RMB	122 (45.0)	
≥5000 RMB	88 (32.5)	
Type of infertility		
Primary	128 (47.2)	
Secondary	143 (52.8)	
Duration of infertility (years)		
<3	158 (58.3)	
≥3	113 (41.7)	
Causes of infertility		
Female factors	91 (33.6)	
Male factors	52 (19.2)	
Both	35 (12.9)	
Unclear	93 (34.3)	
Current treatment regimen		
AI	47 (17.3)	
IVF-ET	148 (54.6)	
ICSI	50 (18.5)	
PGT	26 (9.6)	
Treatment cycle		
1	178 (65.7)	
2 or more	93 (34.3)	

Abbreviations: AI, artificial insemination; IVF-ET, in vitro fertilization and embryo transfer; ICSI, intracytoplasmic sperm injection; PGT, preimplantation genetic testing.

**Table 2:** Comparison of depression, dyadic coping and fertility quality of life scores in infertile couples.

	Wife Mean (SD)	Husband Mean (SD)	r	d	95%CI		Z/*t*	*p*
					Lower	Upper		
Depression	4.45 (4.18)	4.51 (4.20)	0.298 **	-	-	-	−0.149	0.882
DC	112.38 (15.94)	111.62 (16.63)	0.421 **	0.76 (17.54)	−1.334	2.861	0.717	0.474
FertiQoL	66.83 (13.44)	68.43 (13.38)	0.414 **	−1.60 (14.52)	−3.339	0.135	−1.815	0.071

Notes: ** *p* < 0.01; Z, Wilcoxon signed-rank test; *t*, paired *t*-test. Abbreviations: DC, dyadic coping; FertiQoL, fertility quality of life.

**Table 3:** Indicators for each latent profile of dyadic coping among infertile couples undergoing ART.

Model	Class Sample Size	AIC	BIC	aBIC	Entropy	LMRT	BLRT	Proportion (%)
1	C1 = 271	5499.524	5571.567	5508.153	-	-	-	-
2	C1 = 139 C2 = 132	4899.589	5011.255	4912.963	0.819	0.5093	<0.001	0.51/0.49
3	C1 = 107 C2 = 41 C3 = 123	4670.570	4821.859	4688.689	0.866	0.0128	<0.001	0.40/0.15/0.45
**4**	**C1 = 42** **C2 = 62** **C3 = 88** **C4 = 79**	**4510.089**	**4701.002**	**4532.955**	**0.869**	**0.0165**	**<0.001**	**0.16/0.23/0.32/0.29**
5	C1 = 58 C2 = 9 C3 = 81 C4 = 54 C5 = 69	4420.440	4650.976	4448.051	0.882	0.3494	<0.001	0.21/0.03/0.30/0.20/0.26

Note: AIC = Akaike information criterion; BIC = Bayesian information criterion; aBIC = sample size adjusted BIC; LMRT = Lo–Mendell–Rubin likelihood ratio test; BLRT: bootstrapped likelihood ratio test.

**Table 4:** Multinomial logistic regression analysis.

	Variables	Group	b	OR	95% CI	*p*
1 vs. 4	Household registration (wife)	Rural	1.415	4.117	1.331–12.735	0.014
		Urban *				
	Household registration (husband)	Rural	−1.484	0.227	0.080–0.646	0.005
		Urban *				
	Type of infertility	Primary	−1.048	0.350	0.146–0.844	0.019
		Secondary *				
2 vs. 4	Monthly household income per capita	<3000 RMB	1.336	3.805	1.250–11.584	0.019
		3000–4999 RMB	0.926	2.524	1.041–6.120	0.041
		≥5000 RMB *				
	Duration of infertility	<3 years	−1.094	0.335	0.159–0.704	0.004
		≥3 years *				
1 vs. 2	Household registration (husband)	Rural	−1.090	0.336	0.116–0.975	0.045
		Urban *				
	Type of infertility	Primary	−1.279	0.278	0.110–0.703	0.007
		Secondary *				
3 vs. 2	Duration of infertility	<3 years	0.748	2.112	1.053–4.234	0.035
		≥3 years *				

Note: * is the reference group.

**Table 5:** Subgroup comparisons of depression and fertility quality of life among infertile couples undergoing ART.

Profiles	n (%)	Wife		Husband	
		Depression	FertiQoL	Depression	FertiQoL
Class 1	42 (16%)	3.48 ± 3.20	69.23 ± 12.38	6.10 ± 4.93	64.12 ± 10.67
Class 2	62 (23%)	6.63 ± 4.64	61.74 ± 12.08	5.39 ± 4.41	61.68 ± 14.54
Class 3	88 (32%)	4.88 ± 3.98	62.89 ± 13.58	4.53 ± 3.68	67.96 ± 11.45
Class 4	79 (29%)	2.80 ± 3.63	73.92 ± 11.53	2.94 ± 3.66	76.53 ± 11.63
χ^2^		33.88	50.05	20.97	58.95
*p*		0.000	0.000	0.000	0.000
Pairwise differences		Class 4 < 2 and 3; Class 2 > 1, 3 and 4; Class 3 > 1	Class 4 > 1 > 3 = 2	Class 4 < 1, 2 and 3; Class 1 > 3 and 4	Class 4 >1, 2 and 3; Class 3 > 2

Note: Scores are displayed in mean ± SD. Class 1: high-coping wife and low-coping husband group, Class 2: low dyadic coping group, Class 3: medium dyadic coping group, Class 4: high dyadic coping group.

## Data Availability

The datasets generated and analyzed during the current study are not publicly available because the data are also part of an ongoing study; however, the datasets are available from the corresponding author upon reasonable request.

## Supplementary Materials

The following supporting information can be downloaded at https://www.mdpi.com/article/10.3390/healthcare14081031/s1: Table S1. Attribution probabilities for each latent profile of subjects; Table S2. Differences in the demographic and clinical characteristics among the latent profiles.
