# Supplementary material for: Heterogeneity in Dyadic Coping Among Infertile Couples and Its Association with Depression and Fertility Quality of Life: A Latent Profile Analysis
Source: Healthcare (Basel). 2026 Apr 14;14(8):1031. doi: 10.3390/healthcare14081031 (PMC13116404; doi:10.3390/healthcare14081031)
Supplement: Supplementary file 1 [file healthcare-14-01031-s001.zip › healthcare-4201088-supplementary.pdf]

Table S1. Attribution probabilities for each latent profile of subjects.

Class	1	2	3	4
Profile 1	0.930	0.006	0.049	0.015
Profile 2	0.017	0.930	0.053	0.000
Profile 3	0.022	0.035	0.919	0.025
Profile 4	0.009	0.000	0.048	0.943

Table S2. Differences in the demographic and clinical characteristics among the latent profiles.

Variables		Class 1	Class 2	Class 3	Class 4	*F*/χ^2/*H*	*P*
Age (years)							
wives	32.08±4.71(21-47)	32.95±4.40	31.81±4.65	31.60±4.96	32.35±4.65	0.940[a]	0.422
husbands	32.96±4.54(22-48)	33.86±4.17	32.48±4.29	32.97±4.83	32.85±4.60	0.789[a]	0.501
Years of marriage	5.71±3.99(1-23)	6.10±4.00	5.56±3.51	6.22±4.60	5.04±3.56	3.988[c]	0.263
Household registration (wives)						6.429[b]	**0.093**
urban	77(28.4)	10	15	21	31		
rural	194(71.6)	32	47	67	48		
Household registration (husbands)						9.254[b]	**0.026**
urban	74(27.3)	18	16	16	24		
rural	197(72.7)	24	46	72	55		
Education level (wives)						8.619[b]	**0.035**
High school and below	104(38.4)	17	26	41	20		
College and above	167(61.6)	25	36	47	59		
Education level (husbands)						3.165[b]	0.367
High school and below	126(46.5)	17	32	45	32		
College and above	145(54.5)	25	30	43	47		
Employment status (wives)						7.360[b]	**0.061**
Full time	117(43.2)	16	25	32	44		
Part time	154(56.8)	26	37	56	35		
Employment status (husbands)						0.196[b]	0.978

Full time	122(45.0)	20	27	39	36		
Part time	149(55.0)	22	35	49	43		
Monthly household income per capita						17.300[b]	**0.008**
＜3000 RMB	61(22.5)	13	17	21	10		
3000-4999 RMB	122(45.0)	12	34	41	35		
≥5000 RMB	88(32.5)	17	11	26	34		
Type of infertility						9.931[b]	**0.019**
Primary	128(47.2)	11	31	42	44		
Secondary	143(52.8)	31	31	46	35		
Duration of Infertility (years)						10.065[b]	**0.018**
＜3	158(58.3)	23	27	53	55		
⩾3	113(41.7)	19	35	35	24		
Causes of infertility						6.437[b]	0.695
Female factors	91(33.6)	13	20	35	23		
Male factors	52(19.2)	6	12	17	17		
Both	35(12.9)	8	5	10	12		
unclear	93(34.3)	15	25	26	27		
Current treatment regimen						9.818[b]	0.365
AI	47(17.3)	5	14	9	19		
IVF-ET	148(54.6)	22	32	55	39		
ICSI	50(18.5)	10	9	16	15		

PGT	26(9.6)	5	7	8	6		
Treatment cycle						4.510[b]	0.211
1	178(65.7)	32	39	52	55		
2 or more	93(34.3)	10	23	36	24		

Note: a, one-way ANOVA; b, chi-squared test; c, Kruskal–Wallis H test.

Abbreviations: AI, artificial insemination; IVF-ET, in vitro fertilization and embryo transfer; ICSI, intracytoplasmic sperm injection; PGT, preimplantation genetic testing.
